# Prerequisites, barriers and opportunities in care for Q-fever patients: a Delphi study among healthcare workers

**DOI:** 10.1186/s12913-023-09269-y

**Published:** 2023-03-31

**Authors:** Iris M. Brus, Inge Spronk, Juanita A. Haagsma, Vicki Erasmus, Annemieke de Groot, Alfons G. M. Olde Loohuis, Madelon B. Bronner, Suzanne Polinder

**Affiliations:** 1 Department of Public Health, Erasmus University Medical Center, P.O. Box 2040, 3000CA Rotterdam, the Netherlands; 2 Q-Support, ‘s Hertogenbosch, the Netherlands

**Keywords:** Q-fever; Q-fever fatigue syndrome, Chronic Q-fever, Q-fever care, Quality of care

## Abstract

**Background:**

Q-fever is a zoonotic disease that can lead to illness, disability and death. This study aimed to provide insight into the perspectives of healthcare workers (HCWs) on prerequisites, barriers and opportunities in care for Q-fever patients.

**Methods:**

A two-round online Delphi study was conducted among 94 Dutch HCWs involved in care for Q-fever patients. The questionnaires contained questions on prerequisites for high quality, barriers and facilitators in care, knowledge of Q-fever, and optimization of care. For multiple choice, ranking and Likert scale questions, frequencies were reported, while for rating and numerical questions, the median and interquartile range (IQR) were reported.

**Results:**

The panel rated the care for Q-fever patients at a median score of 6/10 (IQR = 2). Sufficient knowledge of Q-fever among HCWs (36%), financial compensation of care (30%) and recognition of the disease by HCWs (26%) were considered the most important prerequisites for high quality care. A lack of knowledge was identified as the most important barrier (76%) and continuing medical education as the primary method for improving HCWs’ knowledge (76%). HCWs rated their own knowledge at a median score of 8/10 (IQR = 1) and the general knowledge of other HCWs at a 5/10 (IQR = 2). According to HCWs, a median of eight healthcare providers (IQR = 4) should be involved in the care for Q-fever fatigue syndrome (QFS) and a median of seven (IQR = 5) in chronic Q-fever care.

**Conclusions:**

Ten years after the Dutch Q-fever epidemic, HCWs indicate that the long-term care for Q-fever patients leaves much room for improvement. Facilitation of reported prerequisites for high quality care, improved knowledge among HCWs, clearly defined roles and responsibilities, and guidance on how to support patients could possibly improve quality of care. These prerequisites may also improve care for patients with persisting symptoms due to other infectious diseases, such as COVID-19.

**Supplementary Information:**

The online version contains supplementary material available at 10.1186/s12913-023-09269-y.

## Background

Q-fever is a zoonotic disease that is prevalent worldwide and that can lead to illness, hospitalization, disability and death [1, 2]. Q-fever is transmitted to humans by inhaling aerosols containing the *Coxiella burnettii* bacteria from birth by-products of domestic animals [1, 3]. Approximately 40% of Q-fever patients experience symptoms. Half of them have mild flu-like symptoms, whereas the other half experience more severe symptoms, such as high fever, pneumonia and hepatitis [4–6].

Between 2007 and 2010, the largest Q-fever outbreak ever reported worldwide occurred in the Netherlands; estimations indicate that over 50,000 individuals were infected with Q-fever [7–9]. After the first outbreak in 2007, the annual number of notified cases rapidly rose and increased tenfold between 2007 and 2009 [7]. Public health authorities were not prepared for a large Q-fever outbreak. Only limited knowledge and evidence on the impact, control measures, identification, treatment and care was available at the time of the outbreak [7, 10, 11].

Q-fever can lead to long-term health consequences, namely Q-fever fatigue syndrome (QFS) and chronic Q-fever [2, 4]. Recent studies estimate that QFS occurs in approximately 20% of Q-fever patients [2]. The clinical manifestation of QFS consists of fatigue persisting for at least 6 months which can be accompanied by a wide range of other symptoms, such as headache, muscle and joint pain and mental health problems [2, 12]. In addition, an estimated 1–2% of infected patients develop chronic Q-fever, which can occur months or years after the initial infection [1, 13]. Common clinical manifestations of chronic Q-fever are endocarditis and infection of aneurysms or vascular prostheses, although the presentation varies [13]. Untreated chronic Q-fever has a high mortality rate of 60% [14].

Both chronic Q-fever and QFS have a substantial impact on the lives of patients. Reukers et al. found that even 5–9 years after acute infection, the quality of life and social functioning of patients with chronic Q-fever and QFS is significantly lower than in the general population and also significantly lower compared to patients with another chronic illness [15]. Bronner et al. support these results and found that the vast majority of patients experienced health problems 10 years after infection. Furthermore, approximately 40% stopped working permanently and over 25% experienced problems with social participation due to their illness [16].

Bronner et al. also found that patients with QFS and chronic Q-fever consulted a median number of 6 different healthcare providers [16]. In comparison, earlier research found that patients with chronic disease in the Netherlands consult a mean of 4.5 different healthcare providers [17]. The majority of patients (75%) was unsatisfied with the overall quality of care for Q-fever. According to patients, the most important barriers were the lack of knowledge of healthcare workers (HCWs), not feeling heard or understood and the lack of availability of services [16].

Currently there is no high quality evidence nor consensus regarding the optimal treatment for QFS [2, 18]. After the large Dutch epidemic, out-patient follow-up care was given to Q-fever patients experiencing prolonged symptoms. However, there was no standardization of the post-infection care provided [10].

To improve the quality of care, insight into barriers and facilitators for the care for Q-fever patients from the perspective of HCWs is necessary. HCWs have the most practical understanding of problems surrounding Q-fever care and are ultimately the ones directly involved in efforts to improve the quality of care. These insights are not only required for improving Q-fever care, but are also relevant for the care for patients who experience long-term sequelae of other infectious diseases. The recent COVID-19 pandemic highlights the global threat infectious diseases pose to public health [19]. As with Q-fever, a significant proportion of COVID-patients experience persisting symptoms months to years after initial infection, usually labelled long COVID or post-COVID-19 condition [20]. The care for long COVID appears to present similar challenges as Q-fever since the clinical manifestation varies considerably and consists of a wide range of symptoms [21, 22]. Thus, insights into the barriers and facilitators of care for Q-fever can also be valuable to improve care for patients with persisting post-infections symptoms as a result of other infectious diseases, such as COVID-19.

The aims of this study were to identify (1) prerequisites for high quality care for Q-fever patients according to HCWs; (2) barriers and facilitators that HCWs experience in the care for Q-fever patients; (3) how these barriers can be overcome; and (4) how care for Q-fever patients should be organized according to HCWs.

## Methods

### Study design

Between February and May 2019, an online two-round Delphi study was conducted among a panel of HCWs in the field of Q-fever in the Netherlands. The Delphi technique is a group facilitation technique that consists of multiple rounds of questionnaires [23]. The purpose is to systematically collect and combine opinions and judgements from a panel of experts on issues on which there is contradictory or insufficient information. Responses of experts are summarized between rounds and used to compose subsequent questionnaires. By anonymously providing information on the answers of the panel participants are able to consider and compare their answers to other experts [23, 24]. To the best of our knowledge, there are currently no studies on the perspectives of HCWs on Q-fever care. Due to the scarcity of previous knowledge on this topic, the Delphi technique was considered the most appropriate method. Applying this method allows for the evaluation of complex issues on which there is scarce information, like Q-fever care, and is especially useful in the explorative phases [25].

### Panel participants

The expert panel participants were selected based on their role in the care for Q-fever patients. They were either directly involved in care and treated Q-fever patients or they were indirectly involved, for example through healthcare policy or Q-fever research. We invited HCWs that were member of the network of Q-support, which is a national center of expertise for Q-fever that supports and advises patients and professionals. The network of Q-support consisted of HCWs who were directly involved in Q-fever care, and most of them were working in regions with high infection rates during the Q-fever epidemic. The Principal Investigator from the Erasmus MC contacted these HCWs via email to inform them and invite them to the Delphi study. In addition, the Principal Investigator contacted HCWs, policy makers and researchers with experience with and/or expertise of Q-fever care that were not part of the network of Q-support from hospitals, research institutes and national working groups, including those tasked with the development of national Q-fever guidelines. Participants received written information regarding the purpose of the study and what it entailed. They were also encouraged to invite colleagues and their own network to participate. Snowball sampling was thus used to reach additional participants [26]. Online informed consent was provided by all participants before participating in the Delphi study.

### Questionnaires

The Delphi study took place following a large scale survey among Q-fever patients [16]. The results from this previous study, as well as other available literature, were used as a starting point for the topics of the questionnaires. Subsequently, input and feedback from experts was collected in two phases. First, input was gathered through a meeting with representatives from Q-support and Q-uestion, which is the patient organization for Q-fever patients in the Netherlands. Second, input was gathered through a meeting with two internists and a doctoral researcher from the Radboud UMC Q-fever Center of Expertise. The Radboud UMC Q-fever Center of Expertise is a collaboration between several departments within the Radboud University Medical Center specialized in treating patients with Q-fever.

The first round online questionnaire contained information about the study purpose and questions on participant characteristics and experience with care for Q-fever patients, knowledge of Q-fever, satisfaction with the provided care, the most optimal care, and collaboration in care. The first round consisted of open ended questions and multiple choices questions. Based on the answers of the first round, a questionnaire was developed for the second round, which allowed for more detailed questions on the topics that were identified in the first round. The answers of four open questions from the first round questionnaire on (1) prerequisites for high quality care, (2) barriers in care, (3) facilitators in care and (4) methods for knowledge improvement were coded and categorized by two independent researchers in order to compose ranking questions for the second round. The second round questionnaire contained information about the study purpose and consisted of ranking questions (from least important to most important), multiple choice questions and questions with 6-point Likert scales (ranging from 1 = strongly agree to 6 = strongly disagree), in addition to several open questions. After one month and a maximum of four reminders the responses were summarized. This study was executed using open-source LimeSurvey software [27].

### Data analysis

Participants who completed at least the first questionnaire round were included in this study. For multiple choice questions, ranking questions and Likert scale questions, frequencies were reported. For rating questions and numerical questions, the median and interquartile range (IQR) were reported due to non-normal distribution of the data. To determine the association between the assessment of personal knowledge and the assessment of general knowledge of HCWs, Spearman’s correlations were used [28]. Data analyses were conducted using SPSS version 25.0 (IBM Corp., Armonk, NY, USA).

## Results

In this section, we first describe the panel characteristics. Second, the current state of Q-fever care is discussed. Third, the prerequisites, barriers and facilitators for high quality care are presented, and one of the main barriers is discussed in more detail. Lastly, we describe how HCWs believe care for Q-fever patients should be organized.

### Panel characteristics

A total of 94 HCWs in the field of Q-fever care participated in the first Delphi round, of whom 86% (*n* = 81) participated in the second round. About half was female (53%), and the median age was 52.0. The panel consisted of a wide range of professions (Table 1). The median number of years in their profession was 15.0. The majority of the panel (63%) was directly involved in care and had treated Q-fever patients, while the remaining 37% were indirectly involved in Q-fever care. Respectively 53% and 42% of the panel had treated patients with QFS and chronic Q-fever. Medical specialists were most often the treating physician of patients with QFS and chronic Q-fever patients (70%; 81%), followed by physio- and occupational therapists (40%; 55%) and general practitioners (38%; 20%) (Additional file 1: Supplementary Table S1).

**Table 1:** Characteristics of expert panel

	**1**^**st**^** round** (*n* = 94)	**2**^**nd**^** round** (*n* = 81)
**Gender**, n (%)		
Female	50 (53.2)	43 (53.1)
Male	44 (46.8)	38 (46.9)
**Age**, median (IQR)	52.0 (22.3)	53.0 (23.5)
**Profession**, n (%)		
Internist	12 (12.7)	9 (11.1)
Physiotherapist	12 (12.7)	12 (14.8)
Other medical specialist (e.g. cardiologist, pathologist)	11 (11.7)	10 (12.3)
General practitioner	11 (11.7)	9 (11.1)
Occupational therapist	11 (11.7)	10 (12.3)
Policymaker	10 (10.6)	9 (11.1)
Researcher	9 (9.6)	9 (11.1)
Occupational physician	5 (5.3)	4 (4.9)
Psychologist	5 (5.3)	2 (2.5)
Alternative practitioner	3 (3.2)	3 (3.7)
Insurance physician	2 (2.1)	1 (1.2)
Other^a^	3 (3.2)	3 (3.7)
**Years in profession**, median (IQR)	15.0 (20.0)	16.0 (21.5)
**HCW**^b^ **who treated patients**, n (%)		
Q-fever	59 (62.8)	50 (61.7)
QFS	50 (53.2)	41 (50.6)
Chronic Q-fever	39 (41.5)	35 (43.2)

^a^The category ‘other’ included a nurse (*n* = 1), a physician assistant (*n* = 1) and a social worker (*n* = 1)

^b^*HCW* Healthcare worker

### Current state of Q-fever care

The panel rated the care for Q-fever patients in general at a median score of 6/10 (IQR = 2). Researchers and policymakers rated the care highest at a 7/10 (IQR = 1), medical specialists gave a median score of 6.5/10 (IQR = 2) and general practitioners, physio- and occupational therapists and other HCWs all rated the care at a 6/10 (IQR = 2). HCWs who treated patients with QFS or chronic Q-fever were also asked how satisfied they were with the care they can provide for these two patient groups. Overall, they rated their satisfaction with care at a 7/10 (IQR = 2) for QFS and an 8/10 (IQR = 1) for chronic Q-fever. General practitioners were least satisfied, rating it at a 5/10 (IQR = 3) for QFS and a 6/10 (IQR = 2) for chronic Q-fever. Medical specialists were most satisfied with their care for chronic Q-fever (8/10, IQR = 1), however, they were less satisfied with their care for QFS (median = 6/10, IQR = 3). Physio- and occupational therapists rated their satisfaction at a 7/10 (IQR = 1–2) for both QFS and chronic Q-fever.

HCWs collaborated with a median of 4 (IQR = 4) healthcare providers in the care for Q-fever patients. Most of them collaborated with general practitioners (60%) and Q-support (50%). HCWs rated the collaboration with healthcare providers in the care for Q-fever patients at a median score of 7/10 (IQR = 2). Across professions, medical specialists rated the collaboration highest (median = 8/10 (IQR = 2)), while all others rated the collaboration at a median score of 6/10 (IQR = 1–2).

### Prerequisites, facilitators and barriers for high quality care for Q-fever patients

HCWs reported many prerequisites for high quality care (Table 2). The most frequently mentioned were: sufficient knowledge of Q-fever among HCWs (36%), financial compensation of care (30%) and recognition of the disease by HCWs (26%).

**Table 2:** Ranking of reported prerequisites for high quality care, showing the percentage of HCWs who placed prerequisite in top 3

Prerequisites	Top 3
Sufficient knowledge of Q-fever among HCWs	36%
Financial compensation of care	30%
Recognition of the disease by HCWs	26%
Up-to-date information for HCWs	22%
Accessible consultation structure with centers of expertise	22%
Multidisciplinary approach to treatment	21%
Treatment guidelines/protocols	20%
High quality diagnostics	20%
More scientific research	18%
Knowledge exchange among HCWs	18%
High quality specialized care and expert centers	10%
Concentration of care, one point of contact	10%
More insight into referral/treatment options	9%
Good regional network of care	8%
High quality medication	8%
Follow-up and continuity of care	7%
National Q-fever network	7%
More treatment options	7%
Clarity about health insurance	3%

Furthermore, five other aspects were also considered to be important by at least 20% of HCWs, including up-to-date information for HCWs, accessible consultation structure with centers of expertise and multidisciplinary approach to treatment.

HCWs considered Q-support, the national center of expertise for Q-fever, and contact with other Q-fever patients the best organized aspects of Q-fever care (62%), followed by the care delivered by centers of expertise and specialists (44%) and the Dutch QFS guideline (25%) (Additional file 1: Supplementary Table S2). The majority of HCWs (76%) who treated QFS patients were familiar with the QFS guideline that was published by the National Institute for Public Health and the Environment. However, 20% of them had not yet applied the guideline in practice. The usefulness of the guideline was rated at a median score of 8/10 (IQR = 2). Although most HCWs were positive about the guideline, several mentioned that the recommendations are targeted at supporting patients, not at curing the disease. As a result, they believed current treatment recommendations for QFS to be insufficient.

According to the panel, a lack of knowledge among HCWs/the disease is not recognized (76%) was the most important barrier for high quality care, followed by unclear/limited scientific evidence for effective treatment (55%) and diagnosis is complex/not always adequate (50%) (Table 3). The majority of the panel (76%) stated that these barriers were different for chronic Q-fever and QFS, mainly because QFS was considered to be more complex and not as clearly defined. In addition, HCWs mentioned that treatment for chronic Q-fever is considered crucial in preventing mortality, while treatment for QFS is targeted at morbidity, resulting in different barriers.

**Table 3:** Ranking of the most important barriers to solve in the care for Q-fever patients, showing the percentage of HCWs who placed barrier in top 3

Barriers	Top 3
Lack of knowledge among HCWs/the disease is not recognized	76%
Unclear/limited scientific evidence for effective treatment	55%
Diagnosis is complex/not always adequate	50%
Lack of high quality guidelines/protocols in healthcare	36%
Treatment is complicated	32%
Lack of collaboration and consultation	27%
Lack of financial compensation for care	18%

**Fig. 1:**
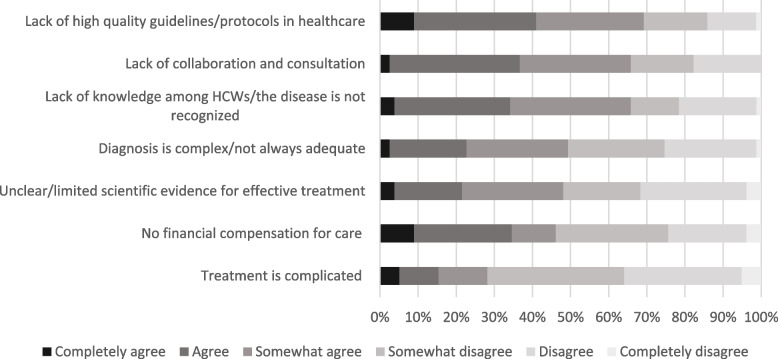
The difficulty of tackling the reported barriers according to HCWs; responses to the statement “This barrier is easy to solve”

The panel was asked whether reported barriers were easy to solve. The most important barrier, lack of knowledge among HCWs/the disease is not recognized, was considered one of the easier barriers to tackle (65%) (Fig. 1). A lack of high quality guidelines/protocols (69%) and a lack of collaboration and consultation (66%) were also considered relatively easy to tackle. The complexity of the treatment (28%) and lack of financial compensation for care (46%) were considered the hardest barriers to tackle.

Within the Dutch healthcare system, care for a specific condition is usually paid through a diagnosis-treatment combination (DBC), meaning that health insurers pay one standard price for the entire care path, not separately for each treatment [29]. Multiple HCWs mentioned that there is not a specific DBC-code for Q-fever fatigue syndrome, leading to insufficient reimbursement of care for Q-fever patients through basic insurance.

### HCWs’ knowledge of Q-fever

Sufficient knowledge of Q-fever was considered the most important prerequisite and a lack of knowledge the most important barrier for high quality care. The panel rated their own knowledge at a median score of 8/10 (IQR = 1). However, they rated the general knowledge level of other HCWs much lower (median = 5/10; IQR = 2) (Additional file 1: Supplementary Table S3). Although the assessment of personal knowledge did not differ between HCWs with and without experience in treating Q-fever patients, HCWs who treated Q-fever patients rated the general knowledge slightly lower than other HCWs: at a median of 5/10 (IQR = 2) and 6/10 (IQR = 2), respectively. The assessment of knowledge appeared to be similar across professions. All professions rated the individual knowledge level at a 7/10 or 8/10 and the general knowledge level at a 5/10 or 6/10, except physio- and occupational therapists, who rated the general knowledge level at a 4/10 (IQR = 2). No observable pattern or correlation was found between the personal knowledge level and general knowledge level of other HCWs (*r* = 0.015; *p* = 0.887).

Continuing medical education (e.g. accredited e-learning, discussion of case studies) was considered the most important method for improving knowledge level (74%), followed by the development of guidelines and protocols (57%) and a good consultation structure and visibility of centers of expertise (44%) (Table 4).

**Table 4:** Ranking of the most important methods for knowledge improvement among HCWs, showing the percentage of HCWs who placed method in top 3

Methods	Top 3
Continuing medical education (accredited e-learning, discussion of case studies at national meetings)	74%
Development of guidelines and protocols	57%
Center of expertise (good consultation structure, sharing experiential knowledge, more visibility)	44%
Scientific research (publications, conferences)	37%
Public attention (national campaign, media attention)	33%
Inclusion in educational curricula (medicine, psychology, general practitioner, physiotherapy)	29%
Professional journals (national and international)	15%

Several HCWs indicated that knowledge improvement should primarily focus on creating more awareness of the long-term effects of Q-fever in order to achieve early diagnosis, as well as on the difference between chronic Q-fever and QFS, also in regions with lower infection rates during the epidemic, as this could lead to more timely and accurate referral of patients. As such, the majority of HCWs (82%) believed that knowledge improvement should mainly focus on primary care. HCWs mentioned that patients are usually first seen in primary care, that the lack of knowledge is most prevalent in primary care and that many patients experience a wide variety of problems. However, others said that extensive education of primary care professionals is not useful as Q-fever is rare and that recognition of the disease is most important. In addition, several HCWs pointed out that knowledge improvement in primary care is particularly relevant for QFS, while knowledge improvement in secondary care is more important for chronic Q-fever, as diagnosis and treatment for the latter condition usually take place in secondary care.

### How care for Q-fever should be organized

According to HCWs, care for Q-fever patients should be provided in a multidisciplinary setting. The median number of healthcare providers needed for optimal Q-fever care was 8 (IQR = 4) for QFS and 7 (IQR = 5) for chronic Q-fever. Most HCWs considered a general practitioner (88%), occupational physician (74%), psychologist (70%), physiotherapist (68%), Radboud UMC Q-fever Center of Expertise (61%) and Q-support (59%) needed in the care for QFS patients. In contrast, a general practitioner (78%), occupational physician (67%), internist (66%), Radboud UMC Q-fever Center of Expertise (60%) and cardiologist (51%) were deemed to be needed in the care for chronic Q-fever. HCWs advised to improve the collaboration between primary and secondary care (42%) and the collaboration between medical care and occupational care/organizations (36%) (Additional file 1: Supplementary Table S4).

HCWs mentioned the importance of having one healthcare professional who coordinates the multidisciplinary care. Most of them indicated that a general practitioner (53%) or a medical specialist working in a center of expertise (30%) should have the ultimate responsibility for QFS care. For chronic Q-fever care, 45% believed a medical specialist working in a center of expertise should have this responsibility, followed by general practitioner (28%) and medical specialist (27%). When asked who should be in charge of the care for QFS and chronic Q-fever in an open format, several HCWs (16% for QFS; 9% for chronic Q-fever) stated that patients should be in charge of their own care.

## Discussion

The results of this study showed that, according to HCWs, the care for Q-fever patients leaves much room for improvement. HCWs indicated many prerequisites for high quality care, of which financial compensation of care and sufficient knowledge of Q-fever among HCWs were most frequently mentioned. However, the panel identified lack of knowledge as the number one barrier in current practice: they indicated that creating more awareness of the long-term effects of Q-fever could lead to more timely and accurate patient referrals. Improving the knowledge level to overcome this barrier is considered to be relatively easy according to HCWs, for example through continuing medical education.

The fact that HCWs considered the lack of knowledge among HCWs to be the most important barrier is in line with the most frequently mentioned barriers by patients [16]. We saw a distinct difference between how HCWs rated their own knowledge and how they rated the knowledge of HCWs in general, which might be explained by the fact that most of the HCWs in our study are experts in the field of Q-fever. These results indicate that knowledge improvement could be an important building block for improving care for Q-fever patients. That most HCWs consider the lack of knowledge relatively easy to tackle seems promising, although this might be due to their belief that others should improve their knowledge.

A lack of knowledge among HCWs has also been identified as a key issue impeding high quality care for patients with other post-infectious syndromes and Myalgic Encephalomyelitis/Chronic Fatigue Syndrome (ME/CFS), a similar condition to QFS [30–32]. Previous research on implementing resources to support the diagnosis and management of ME/CFS showed the difficulty of improving knowledge among general practitioners due to the complexity of such conditions and the low prevalence [33]. HCWs in our study also emphasize these issues, which indicate the importance of tailoring the format and extent of continuing medical education to the needs of HCWs and what they encounter in daily practice. A short and general program, for instance through online modules, aimed at improving knowledge of multiple post-infectious syndromes, including QFS and post-COVID-19 condition, could be offered to a broad audience of HCWs. Awareness and recognition of post-infectious syndromes should be the priority for most HCWs. However, those working with patients in high-risk areas (i.e. high infection rates during Q-fever epidemic) would likely benefit from more extensive education on follow-up steps and treatment of Q-fever patients in a different, more interactive format.

Closely related to the lack of knowledge of Q-fever is the limited and unclear scientific evidence for effective treatments, which the majority of HCWs considered to be another significant barrier. More scientific research and up-to-date information for HCWs were frequently mentioned prerequisites for high quality care. However, over half of HCWs believed that the problem of limited scientific research is not easily tackled. Recent studies also emphasize that there is insufficient evidence on treatment options for Q-fever patients as well as on prognosis and risk factors for a severe disease course [2, 34, 35]. Although the dissemination of existing scientific knowledge will undoubtedly contribute to the quality of Q-fever care, more research on treatment options is also essential for better disease management.

The need for multidisciplinary care also appears to be an important aspect of Q-fever care as HCWs indicate that many healthcare providers from different disciplines should be involved in the care for QFS and chronic Q-fever. The recently updated QFS guideline also emphasizes this: although there is no scientific literature available on the effectiveness of multidisciplinary treatment of QFS patients, the working group who developed the guideline advises healthcare providers to consider the option of referral to a specialized center for multidisciplinary treatment [12]. However, HCWs reported that they currently collaborate with a median of four healthcare providers, so although HCWs acknowledge that the care preferably involves many disciplines, based on the reported collaborations, this ideal seems to be difficult to realize.

One aspect that possibly impedes effective multidisciplinary collaboration is a lack of clear roles and responsibilities of the different healthcare providers [36]. Although the majority of HCWs indicated that a general practitioner should have the ultimate responsibility for QFS care and a medical specialist for chronic Q-fever, quite a large group believed otherwise (47% and 28%, respectively), suggesting some disagreement about the preferred role division between different professions. Nevertheless, in order to enable effective multidisciplinary collaboration, it is essential that HCWs agree and act on their respective roles and responsibilities as a lack of understanding of the roles and responsibilities of others could negatively impact collaboration and lead to a lower quality of care [36–38]. Unfortunately, current guidelines and care pathways provide insufficient guidance on the roles and responsibilities of the different professions involved in the care for these patients, nor on how to accomplish effective multidisciplinary collaboration [12, 39].

In addition to sufficient knowledge among HCWs, up-to-date information for HCWs and a multidisciplinary approach to treatment, HCWs indicated many other prerequisites for high quality care. The large number of prerequisites shown in this study indicates the complexity of Q-fever care and shows that it is unlikely that there is one ready-made solution to improve the quality of care. Furthermore, these prerequisites have implications for several aspects of healthcare. While sufficient knowledge directly concerns HCWs, the condition of financial compensation of care applies to policymakers in healthcare. The high ranking of financial compensation indicates that the problems surrounding Q-fever care – as well as the solutions – do not only lie with HCWs, but that healthcare policy plays a key role.

When comparing ratings of Q-fever care of HCWs to those of Dutch patients, we see a clear discrepancy between how HCWs and patients perceive the quality of care. HCWs in our study rate the care for Q-fever at a median score of 6/10, while in earlier research, Q-fever patients gave a median score of 4/10 [16]. Although the question was formulated similarly for HCWs and patients, these ratings might not be directly comparable, as the interpretation of ‘quality of care’ possibly differs between HCWs and patients. As suggested by Bronner et al., HCWs and patients may have different perceptions of relevant aspects of health [16]. Previous research has shown that HCWs’ and patients’ expectations of care as well as their methods of assessing care differ [40, 41]. Huber et al. found that patients consider many dimensions of health to be important, including mental functions and perception, social participation and daily functioning, while healthcare providers, especially doctors, look at health from a more biomedical viewpoint [42]. These diverging views on health might translate to different perceptions of the quality of Q-fever care. The comparison of satisfaction with care between HCWs and patients should thus be interpreted with caution. However, the difference implies that patients are less satisfied with Q-fever care than HCWs, although both groups indicate that there is much room for improvement and a need to identify barriers in Q-fever care.

HCWs in our study are less satisfied with the care they can provide for QFS than for chronic Q-fever. This contrast is also reflected in patients’ assessments: Bronner et al. reported that patients with QFS rate their care significantly lower than those with chronic Q-fever, at a 3/10 and 6/10, respectively [16]. We hypothesize that the strong dissatisfaction of both HCWs and patients with QFS care compared to chronic Q-fever care may be related to the concept of cure versus care. Cure is aimed at healing and recovery, while care is aimed at limiting the negative effects of a condition on multiple aspects of patients’ lives as much as possible [43–45]. HCWs in this study mentioned that barriers differ as treatment for chronic Q-fever is crucial in preventing mortality, while treatment for QFS is targeted at morbidity. Furthermore, while there are effective treatment options for chronic Q-fever, there is a lack of evidence-based treatment for QFS [39, 46]. Thus, the treatment of chronic Q-fever is mainly focused on cure, whereas the treatment of QFS has a much stronger emphasis on care. Previous research has shown that HCWs experience difficulties when treatment for a condition is care-focused. Qualitative research on dementia care found that physicians struggled with the care for patients when standard pharmacological interventions were no longer effective [44], and research on care for patients with medically unexplained symptoms showed that general practitioners experience frustrations when caring for these patients due to their inability to ‘fix’ the problem [47, 48]. Researchers suggested that communication skill courses as well as increased emphasis on psychosocial aspects during medical training may lead to a more care-oriented attitude [47, 49].

The results from our study and a previous study among Q-fever patients [16] indicates that the same difficulties with cure versus care may apply to QFS care: HCWs and patients acknowledge the poor quality of care, yet improving the quality of care remains challenging. Although the current QFS guideline states the importance of providing patients with support in handling their problems and limitations, it contains little direction on how this should be accomplished [12]. The scarcity of information on care-focused aspects in guidelines has also been identified in previous research [44]. Providing HCWs with more guidance on care-focused aspects of treatment via existing or new clinical practice guidelines may contribute to the challenge of improving the quality of care as well as the satisfaction of both HCWs and patients with the provided care.

Some HCWs mentioned that creating care networks similar to existing networks such as ParkinsonNet could increase the quality of care through improving two important aspects: multidisciplinary collaboration and knowledge. Parkinson, like QFS and chronic Q-fever, is a complex chronic disease that requires the involvement of many different professionals. In the ParkinsonNet model care is concentrated among trained professionals organized in regional networks. Collaboration between professionals is facilitated by a dedicated online platform, which also provides patients with information on access to care and quality of care [50, 51]. Bloem et al. suggest that this model could also improve care for other chronic disorders [51]. Consequently, the ParkinsonNet model could possibly provide tools for further development of clinical practice guidelines and care networks of healthcare providers for care for Q-fever patients.

In light of the current COVID-19 pandemic, it is essential to draw lessons from the challenges of Q-fever care. The immense reach of COVID-19 makes it crucial to provide high quality care to patients, especially to those with long COVID [21]. Following the Dutch Q-fever epidemic, there was no standardization of post-infection care, scarce information on identification and treatment, and the scale of the impact of long-term consequences only became clear in recent years, a decade after the start of the epidemic [2, 10]. Fortunately, there is a great deal of attention for possible long-term sequelae of COVID-19, and the clinical case definition of post-COVID-19 condition by the World Health Organization hopefully facilitates both healthcare research and practice [52]. Nevertheless, our results indicated several aspects that appear to be relevant for providing high quality care that require attention when organizing care for COVID-19, namely: ensuring that HCWs have sufficient knowledge, especially with rapidly emerging new evidence; considering how to structure complex care involving many different disciplines; and concentrating on both cure- and care-focused treatment aspects.

### From research to practice

Knowledge improvement appears to be necessary and could possibly be achieved through educational programs for HCWs, tailored to their specific situation, on post-infectious syndromes, with a focus on creating awareness of long-term effects. This could be supported by creating one national portal for up-to-date information. More effective multidisciplinary collaboration may be attained by clarifying the respective roles and responsibilities of HCWs in guidelines as well as the organization of national or regional networks of HCWs, similar to ParkinsonNet. In addition, a more care-oriented attitude towards treatment may be needed to improve care for Q-fever, especially Q-fever fatigue syndrome, which may be accomplished through more communication skill courses for HCWs focused on discussing and evaluating psychosocial aspects. Clarifying how HCWs can help patients deal with limitations in existing guidelines, for example by specifying topics that should be discussed during consultation as well as possible avenues through which HCWs can provide patients with additional support, could also help to achieve a more care-oriented attitude and improve the quality of care. Although HCWs play an essential role in identifying issues in and improving care, the responsibility for designing and implementing programs to improve Q-fever care through these different avenues primarily lies with policy makers, namely the ministry of Health and national health institutes, possibly supported by centers of expertise.

### Strengths and weaknesses

This study has several strengths. First, we included a large number of participants in this study. Although there is no clear minimum sample size for Delphi studies, the 94 HCWs that participated is a much larger sample than the sample size of 10–18 panel members as recommended by Okoli et al. [53]. Second, we included HCWs from many different professions in order to get a wide range of perspectives. Third, although the long-term consequences for Q-fever patients and their experiences with Q-fever care have been investigated, to the best of our knowledge, the perspectives of HCWs on Q-fever care have not yet been studied. This study makes an important contribution as it not only provides insight into the barriers and facilitators in Q-fever care, but offers tools for improving the quality of care as well.

This study also has some limitations. First, the panel consisted of experts either directly or indirectly involved in Q-fever care, which might paint an overly optimistic picture of the care for these patients. The nature of a Delphi study is that experts express their views on a certain topic, but whether these also reflect views of other HCWs is unknown. From previous research we know that outcomes may differ depending on the group of experts that was consulted [25]. However, we attempted to collect a diversity of opinions by including many HCWs from a variety of disciplines, in order to improve the robustness and validity of the results. Second, several of the themes addressed in this Delphi study have been asked in a very broad, overarching manner, such as the (lack of) knowledge of HCWs and the multidisciplinary aspect of care. This broad approach is due to the exploratory nature of the study and its design. We believe that this first phase was needed, due to the scarce knowledge on this topic, and that the insights gathered in this study are a valuable step towards improving the quality of Q-fever care. However, more in-depth research, possibly through in-depth interviews, into the themes addressed in this study is needed to draw any definitive conclusions and to come up with concrete recommendations. Third, although Delphi-studies are typically completed in two to three rounds, there is no consensus on the number of rounds that should be performed in a Delphi study [25]. However, we do recognize that only two rounds means that we have limited information on the stability of responses between rounds, and additional rounds would be needed to determine consistency of responses and stability of rankings [54].

## Conclusions

Ten years after the Q-fever epidemic HCWs indicate that the care for Q-fever patients still leaves much room for improvement. This exploratory study provides directions for possible avenues to advance the quality of care, namely: improving the knowledge level of HCWs; ensuring that HCWs are aware of and agree on their respective roles and responsibilities to enable effective multidisciplinary collaboration; and providing HCWs with guidance on how to support patients in handling their problems and limitations. More scientific evidence on treatments, prognosis and risk factors; further development and specification of clinical practice guidelines and the implementation of care networks based on existing models for other similarly complex and variable diseases may help achieve these goals and improve the care for patients with persisting post-infectious symptoms as a result of other diseases, such as COVID-19, and are thus valuable in the organization of care for COVID-19 patients.

## Supplementary Information

**Additional file 1: Table S1.** Additional characteristics of HCWs in expert panel directly involved in Q-fever care. **Table S2.** Ranking of best organized aspects in the care for Q-fever patients, showing the percentage of HCWs who placed aspect in top 2 (participants were asked to select at least two aspects). **Table S3.** Score of personal knowledge level and general knowledge level of HCWs according to expert panel. **Table S4.** Collaborations that should be improved in the care for Q-fever, showing the percentage of HCWs who placed collaboration in top 2 (participants were asked to select two collaborations).

## Data Availability

The dataset generated and analysed during the current study is available from the corresponding author on reasonable request.

## Abbreviations

ME/CFS: Myalgic Encephalomyelitis/Chronic Fatigue Syndrome
HCW: Healthcare worker
IQR: Interquartile range
QFS: Q-fever fatigue syndrome
