# Supplementary material for: Prerequisites, barriers and opportunities in care for Q-fever patients: a Delphi study among healthcare workers
Source: BMC Health Serv Res. 2023 Mar 31;23:319. doi: 10.1186/s12913-023-09269-y (PMC10064509; doi:10.1186/s12913-023-09269-y)
Supplement: Supplementary file 1 — Additional file 1: Table S1. Additional characteristics of HCWs in expert panel directly involved in Q-fever care. Table S2. Ranking of best organized aspects in the care for Q-fever patients, showing the percentage of HCWs who placed aspect in top 2 (participants were asked to select at least two aspects). Table S3. Score of personal knowledge level and general knowledge level of HCWs according to expert panel. Table S4. Collaborations that should be improved in the care for Q-fever, showing the percentage of HCWs who placed collaboration in top 2 (participants were asked to select two collaborations). [file 12913_2023_9269_MOESM1_ESM.docx]

**Table S1:** Additional characteristics of HCWs in expert panel directly involved in Q-fever care

|  | **1^st^ round** | **2^nd^ round** |
| --- | --- | --- |
| **Number of QFS patients HCW had treated**, n (%)  <5  5-10  10-20  >20  I don’t know | 21 (42.0)  7 (14.0)  5 (10.0)  15 (30.0)  2 (4.0) | 20 (48.8)  3 (7.3)  4 (9.8)  12 (29.3)  2 (4.9) |
| **Number of chronic Q-fever patients HCW had treated**, n (%)  <5  5-10  10-20  >20  I don’t know | 10 (25.6)  9 (23.1)  4 (10.3)  14 (35.9)  2 (5.1) | 10 (28.6)  9 (25.7)  4 (11.4)  10 (28.6)  2 (5.7) |
| **HCW was the treating physician of QFS patients**, n (%)  Yes  No  I don’t know | 22 (44.0)  21 (42.0)  7 (14.0) | 15 (36.6)  19 (46.3)  7 (17.1) |
| **HCW was the treating physician of chronic Q-fever patients**, n (%)  Yes  No  I don’t know | 21 (53.8)  16 (41.0)  2 (5.1) | 18 (51.4)  15 (42.9)  2 (5.7) |
| **Phase in which HCW provided care for QFS patients**, n (%)  Detection  Diagnosis  Short term (1^st^ 12 months)  Long term (after 1^st^ 12 months)  *Involved in all four phases of care* | 10 (20.0)  17 (34.0)  20 (40.0)  42 (84.0)  *5 (5.3)* | 8 (19.5)  12 (29.2)  14 (34.1)  36 (87.8)  *4 (4.9)* |
| **Phase in which HCW provided care for chronic Q-fever patients**, n (%)  Detection  Diagnosis  Short term (1^st^ 12 months)  Long term (after 1^st^ 12 months)  *Involved in all four phases of care* | 11 (28.2)  18 (46.2)  21 (53.8)  35 (89.7)  *9 (9.6)* | 8 (22.9)  14 (40.0)  17 (48.6)  31 (88.6)  *6 (7.4)* |

**Table S2:** Ranking of best organized aspects in the care for Q-fever patients, showing the percentage of HCWs who placed aspect in top 2 (participants were asked to select at least two aspects)

| **Aspects** | **Top 2** |
| --- | --- |
| Q-support and contact with other patients | 62% |
| Care by centers of expertise and specialists | 44% |
| Guideline for QFS | 25% |
| Public attention for Q-fever | 18% |
| National database of chronic Q-fever | 16% |
| Diagnostics for chronic Q-fever | 16% |
| Set-up of a network of Q-fever healthcare professionals | 10% |
| Good collaboration and possibility of accessible consultation | 9% |

**Table S3:** Score of personal knowledge level and general knowledge level of HCWs according to expert panel

|  | **Personal knowledge** | **General knowledge** |
| --- | --- | --- |
|  | Median (IQR) | Median (IQR) |
| **All experts** | 8 (1) | 5 (2) |
| **Experience with Q-fever** |  |  |
| Treated Q-fever patients | 8 (1) | 5 (2) |
| Not treated Q-fever patients | 8 (1) | 6 (2) |
| **Profession** |  |  |
| General practitioner | 7 (1) | 5 (2) |
| Physio/occupational therapist | 7 (1) | 4 (2) |
| Medical specialist | 8 (1) | 5 (2) |
| Researcher/policy maker | 8 (2) | 5 (2) |
| Other | 7 (2) | 6 (2) |

**Table S4:** Collaborations that should be improved in the care for Q-fever, showing the percentage of HCWs who placed collaboration in top 2 (participants were asked to select two collaborations)

| **Collaborations** | **Top 2** |
| --- | --- |
| Primary care (general practitioner, physiotherapist) and secondary care (medical specialist) | 42% |
| Medical care and occupational care/organizations (occupational physician, inspection authorities) | 36% |
| Primary care and centers of expertise | 21% |
| Healthcare professionals in primary care | 13% |
| Paramedics (occupational therapy, physiotherapy, psychology, mental health care) | 13% |
| Medical care and health insurers | 12% |
| Medical care and the government | 9% |
| Healthcare professionals in secondary care (medical specialists) | 9% |
| Medical care and public health service (Municipal Health Services, preventive care) | 6% |
| Medical care and policymakers/network organizations | 5% |
| Non-medical organizations | 5% |
| Medical care and paramedics | 5% |
| Secondary care and centers of expertise | 5% |
